# Supplementary material for: A temporal sequence of thalamic activity unfolds at transitions in behavioral arousal state
Source: Nat Commun. 2022 Sep 16;13:5442. doi: 10.1038/s41467-022-33010-8 (PMC9481532; doi:10.1038/s41467-022-33010-8)
Supplement: Supplementary file 1 — Supplementary Information [file 41467_2022_33010_MOESM1_ESM.pdf]

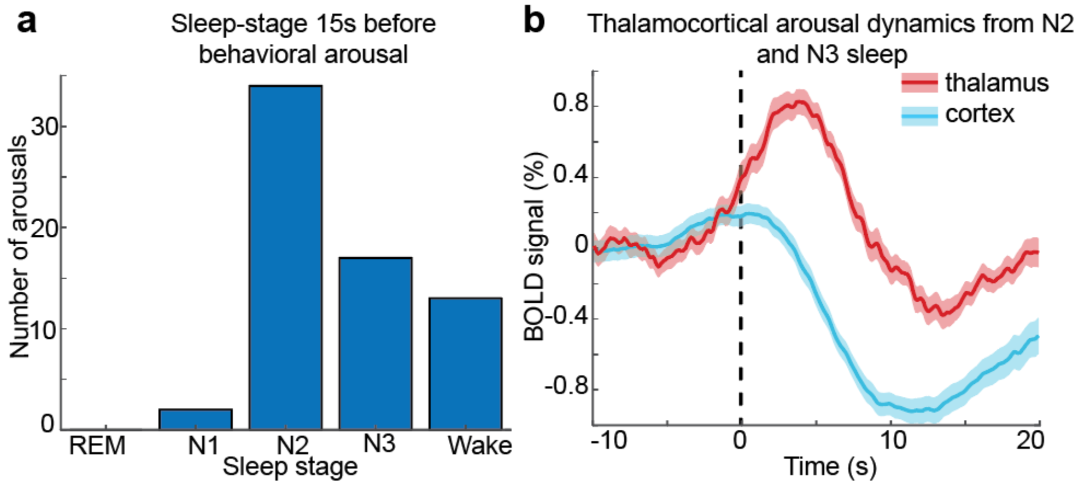

Supplementary Figure 1: a) Sleep stages 15 seconds before each arousal in Experiment 1 demonstrate that most arousals occurred from N2 and N3 sleep. b) When arousals from wake and N1 sleep are excluded, the same thalamocortical dynamics are preserved. Data are presented as mean values and shading represents standard error. Source data are provided in "Fig S1b Source Data" file.

Metric	Per subject	Average	Standard deviation	Minimum, maximum
Number of behavioral arousals	N per subject = (3, 22, 9, 29, 2, 1)	11 behavioral arousals	11.78 behavioral arousals	Min=1, max=29 behavioral arousals
Time between arousals	N/A	287.18 s	479.55 s	Min=24.53 s, 3051.56 s
Rate of arousals	N/A	0.63 arousals per 5 min	1.26 arousals per 5 min	Min=0, Max=7 arousals per 5 min

Supplementary Table 1: Descriptive metrics of behavioral arousals in Experiment 1. Row 1: the number per subject, average, standard deviation, minimum and maximum of the number of behavioral arousals. Row 2: the time between behavioral arousals for functional runs which had more than 1 arousal. Row 3: the rate of arousals per 5 minutes.

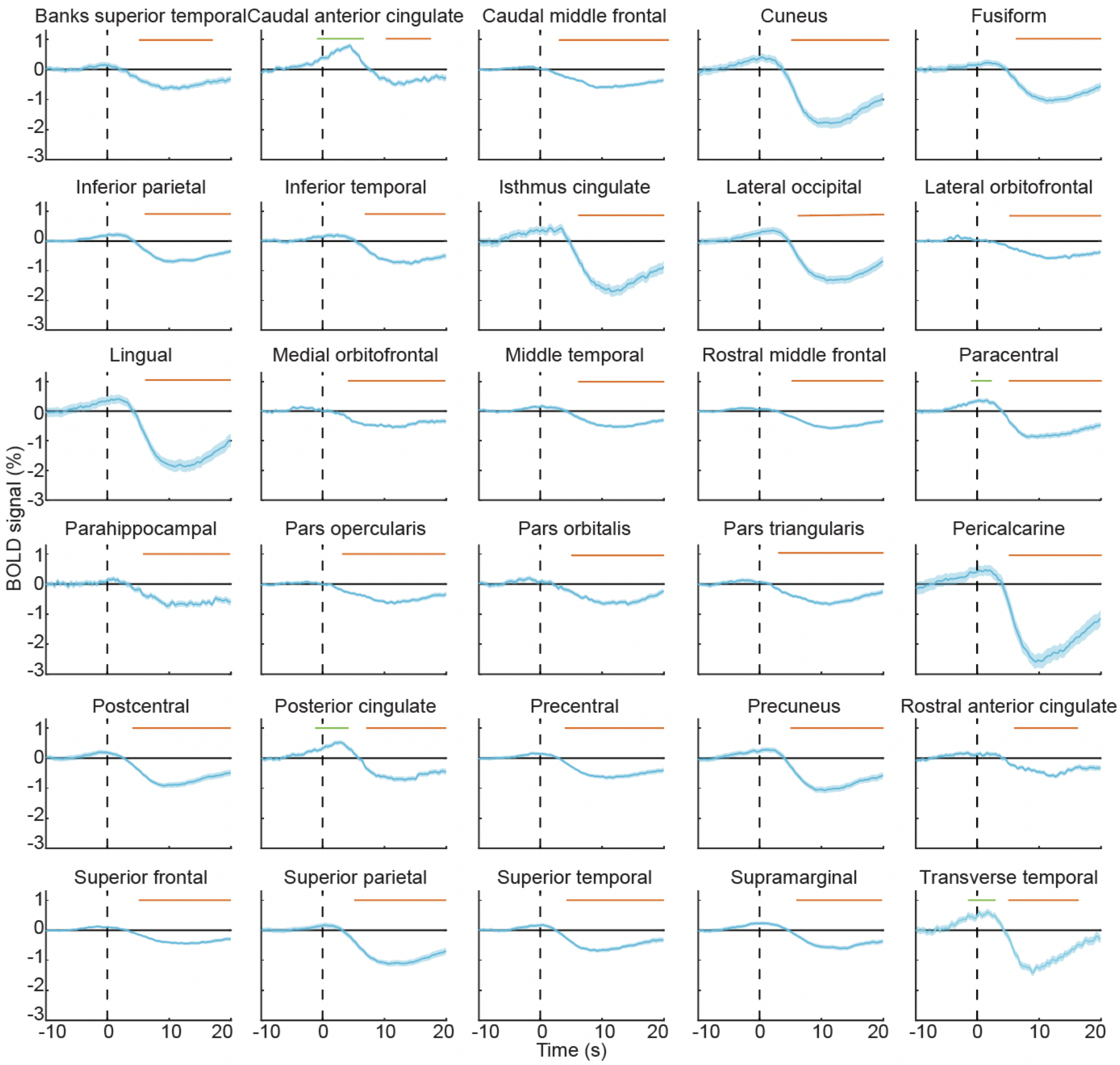

Supplementary Figure 2: All cortical regions at 3T during behavioral arousal (Experiment 1). The vertical black dashed line represents the moment of behavioral arousal. A subset of cortical regions including the caudal anterior cingulate, posterior cingulate, paracentral, and transverse temporal cortices significantly increased during behavioral arousal (green bar). All cortical regions significantly decreased during arousal (orange bar). Data are presented as mean values and shading represents standard error. Source data are provided as “Fig 2 Source Data” file.

Metric	Per subject	Average	Standard deviation	Minimum, maximum
Number of behavioral arousals	N per subject= (1, 9,11, 3, 10, 6, 21, 5, 1, 6, 8, 13, 3)	7.46 behavioral arousals	5.55 behavioral arousals	Min=1, max=21 behavioral arousals
Time between arousals	N/A	218.66 s	274.66 s	Min=20.73 s, max =1333.27 s
Rate of arousals	N/A	0.47 arousals per 5 min	0.93 arousals per 5 min	Min=0, Max=4 arousals per 5 min

Supplementary Table 2: Descriptive metrics of behavioral arousals in Experiment 2. Row 1: the number per subject, average, standard deviation, minimum and maximum of the number of behavioral arousals. Row 2: the time between behavioral arousals in functional runs which had more than one behavioral arousal. Row 3: the rate of arousals per 5 minutes.

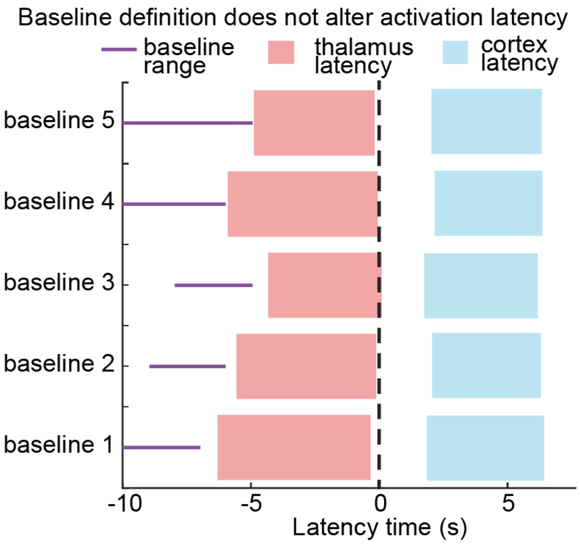

Supplementary Figure 3: The specific baseline range (purple line) does not affect relative thalamic and cortical onset latencies. Activity onset latency is defined by the time the signal reaches 20% of its maximum absolute amplitude from baseline. Thalamus onset latency (red) occurs before behavioral arousal (dashed line), and cortical onset latency occurs afterwards (blue). Shaded boxes represent the 95% confidence intervals.

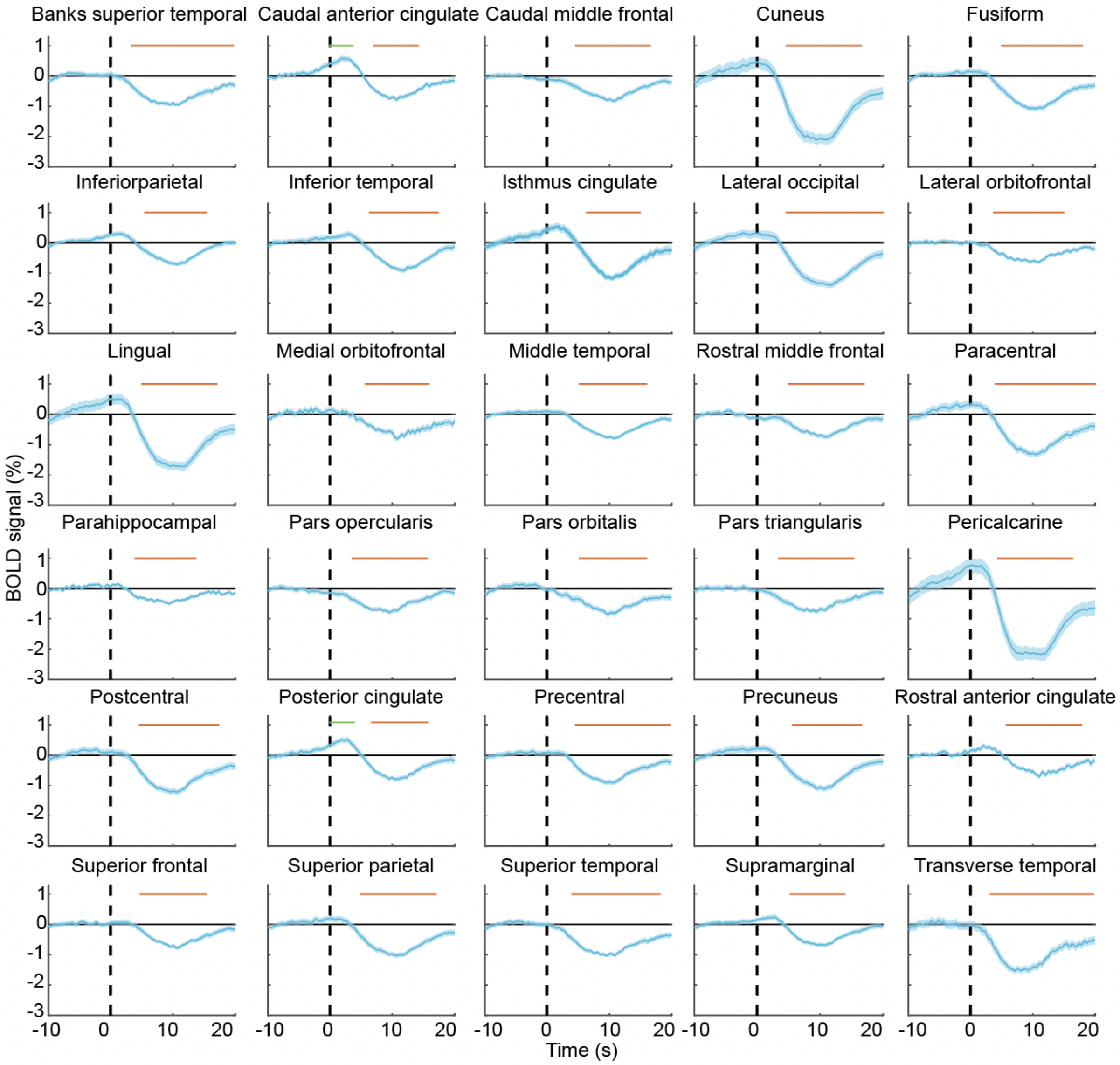

Supplementary Figure 4: All cortical regions at 7T during behavioral arousal (Experiment 2). The vertical black dashed line represents the moment of behavioral arousal. A subset of cortical regions significantly increased during arousal (green bar), including the caudal anterior cingulate, and posterior cingulate. All cortical regions significantly decreased during arousal (orange bar). Data are presented as mean values and shading represents standard error. Source data are provided in “Fig 3 Source Data” file.

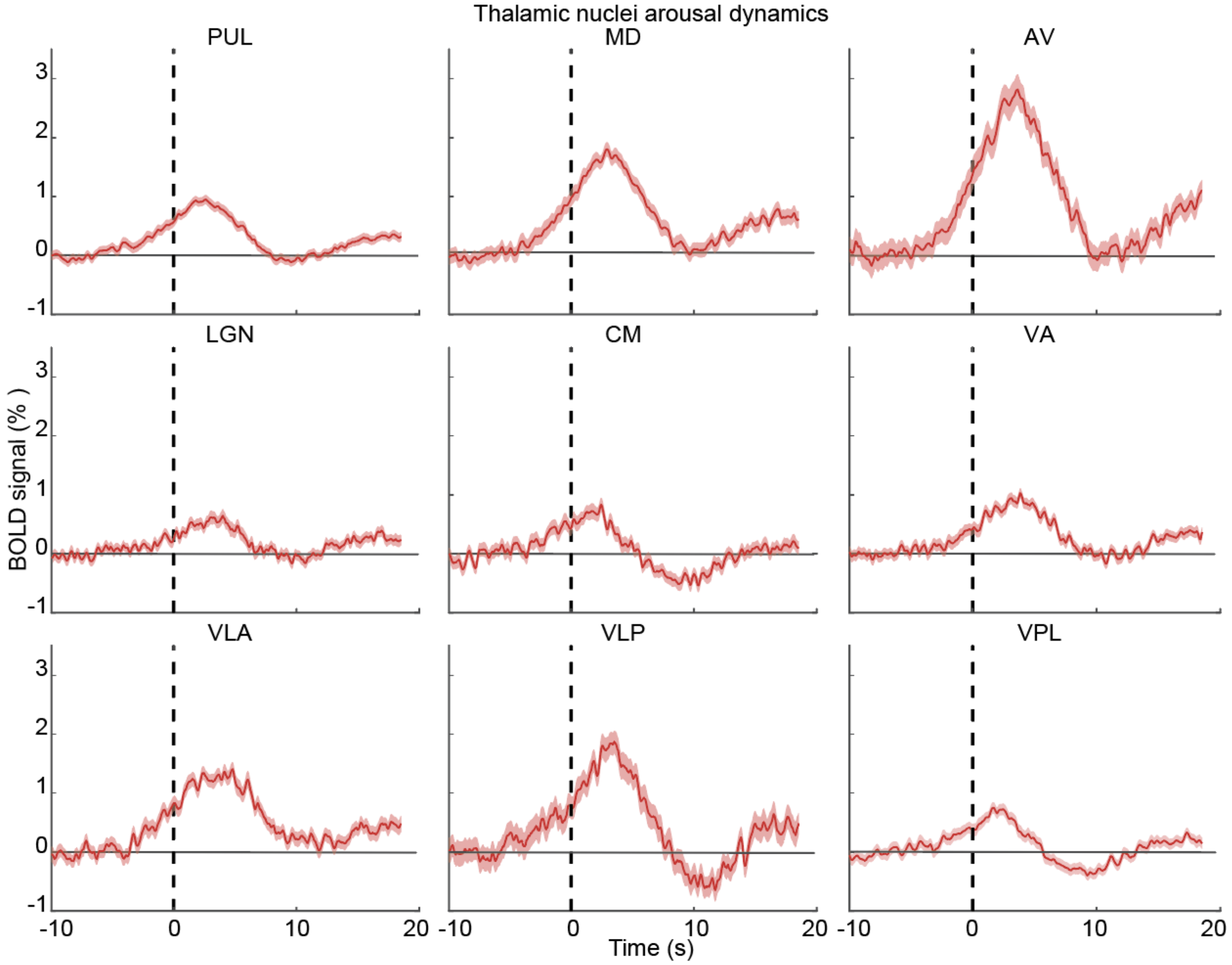

Supplementary Figure 5. Thalamic nuclei activate during behavioral arousal (vertical dashed line). Data are presented as mean values and shading represents standard error. Source data are provided in “Fig 3 Source Data” file.

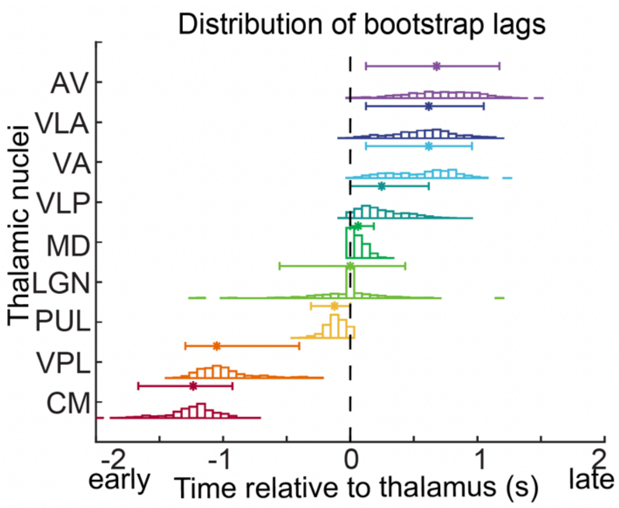

Supplementary Figure 6. The distribution of mean lags calculated in the bootstrap analysis. The maximum value on each histogram's y-axis is 550 (representing number of bootstrap samples; 1000 samples total). The bounded horizontal line represents the 95% confidence interval. The mean lag of the average timeseries (not from bootstrap analysis) is represented by a star. The histograms show non-Gaussian distributions.

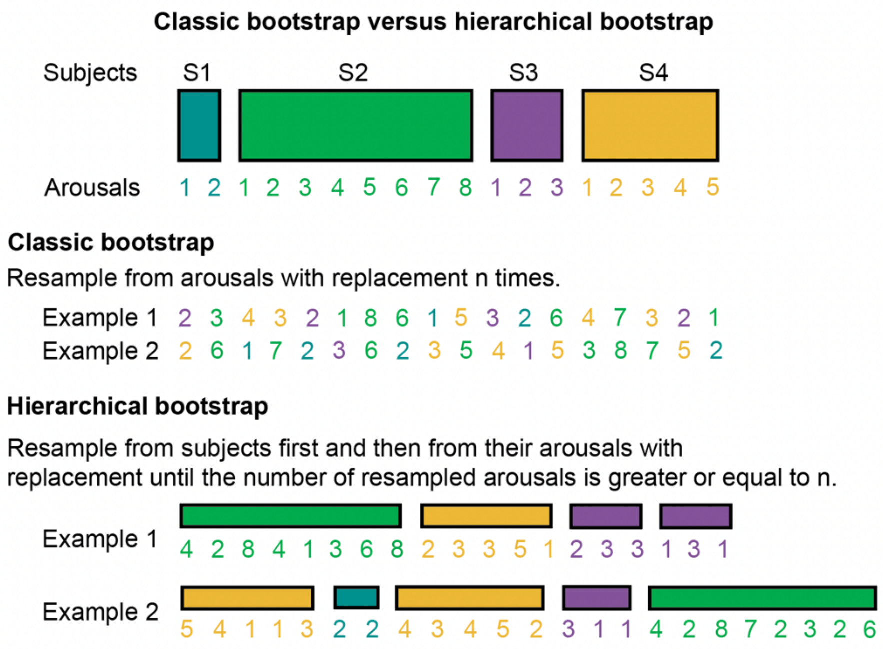

Supplementary Figure 7. Schematic of the procedure for classical and hierarchical bootstrap.

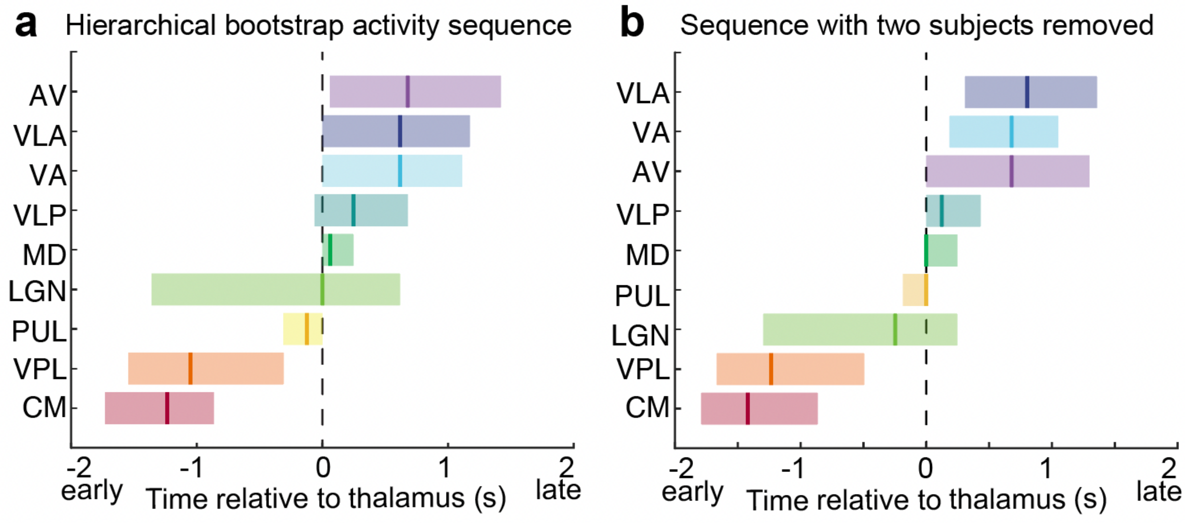

Supplementary Figure 8: a) Lag sequence of thalamic nuclei using a hierarchical bootstrap. Subjects are resampled, and then arousals are resampled to generate the 95% confidence intervals (shaded boxes). Solid line represents the mean lag. b) Lag sequence when excluding the two subjects that had the most arousals, (n=21, 13 arousals). Color represents order in original lag sequence from red to purple. CM and VPL remain significantly early, and VA, AV, and VLA remain significantly later (n=11 subjects, 63 behavioral arousals). Source data are provided as in “Fig S8 Source Data” file.

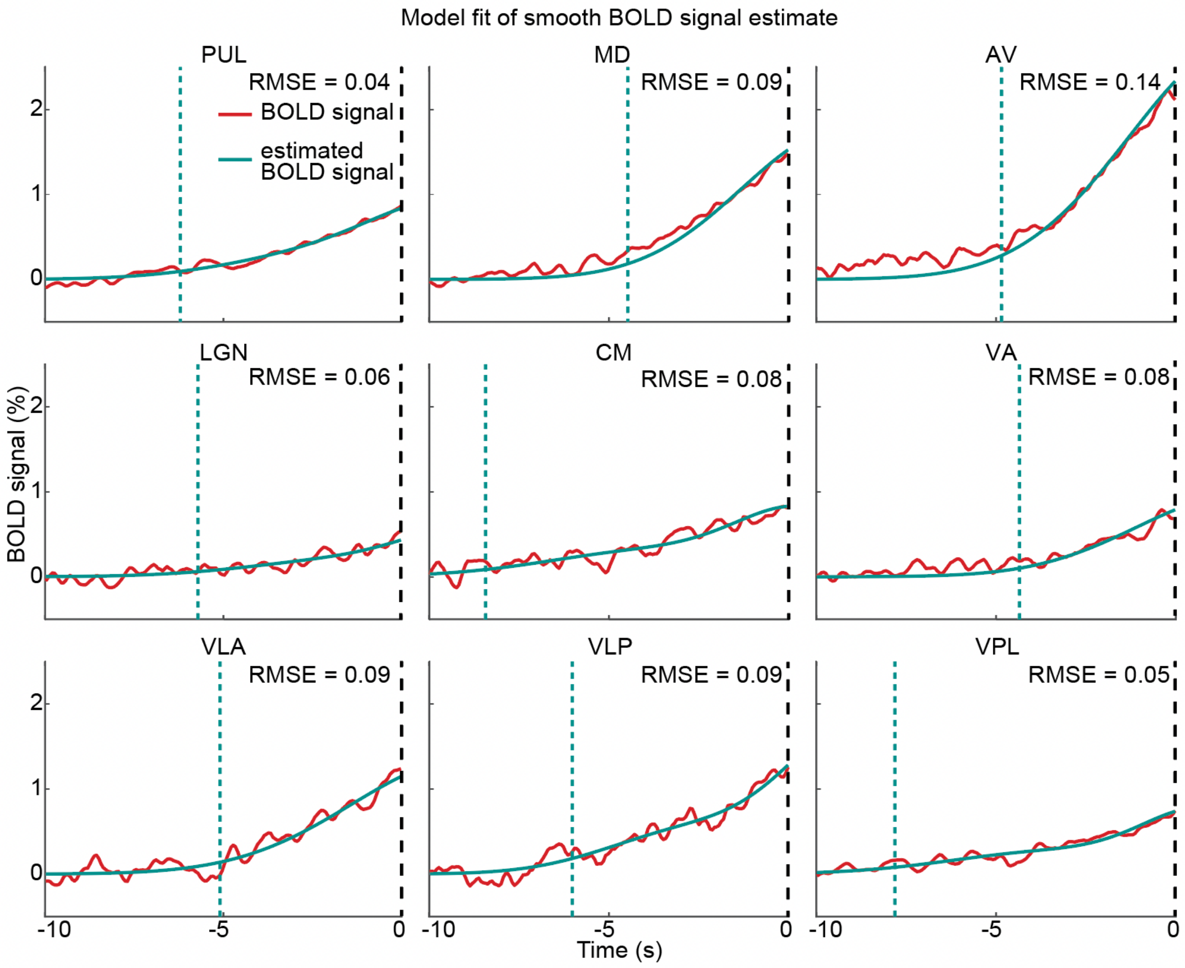

Supplementary Figure 9: Onset times of thalamic nuclei during behavioral arousal. The BOLD signal is in red and the model fit is in teal. The onset time of each thalamic nucleus is represented by the teal arrow. Behavioral arousal is marked by the black dashed line. Source data is provided in “Fig 4 Source Data” file.

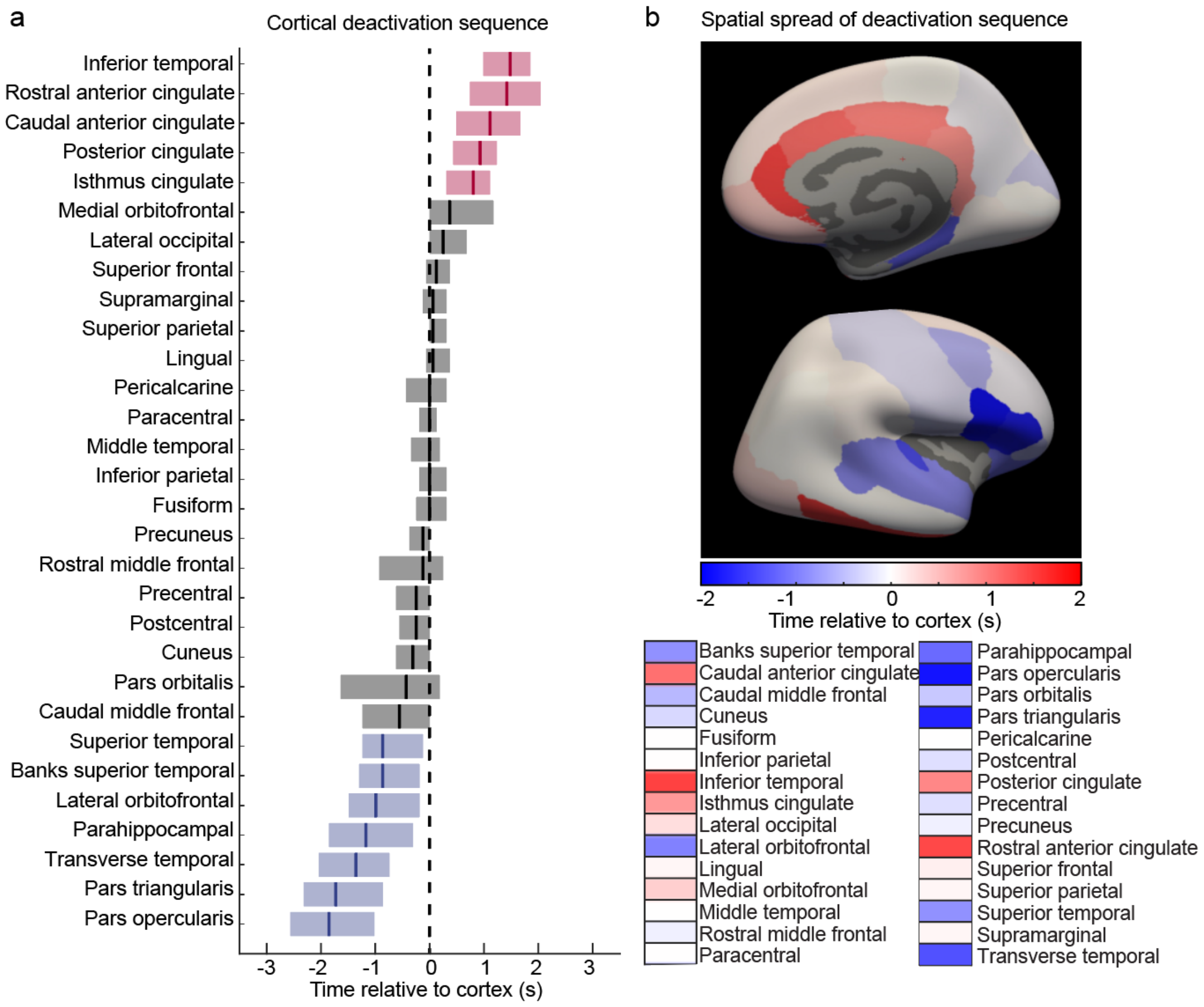

Supplementary figure 10: Cortical lag sequences. a) A sequence of cortical deactivation occurs during behavioral arousal. A subset of cortical regions deactivates earlier (blue) than the global cortical signal (black, dashed line), and a subset deactivates late (red). The mean lag relative to the global cortical signal is denoted by a solid, vertical line. Shading represents 95% confidence interval. b) The spatial spread of cortical lags. Cingulate cortex deactivates later than pars cortex. Source data are provided in “Fig 3 Source Data” file.

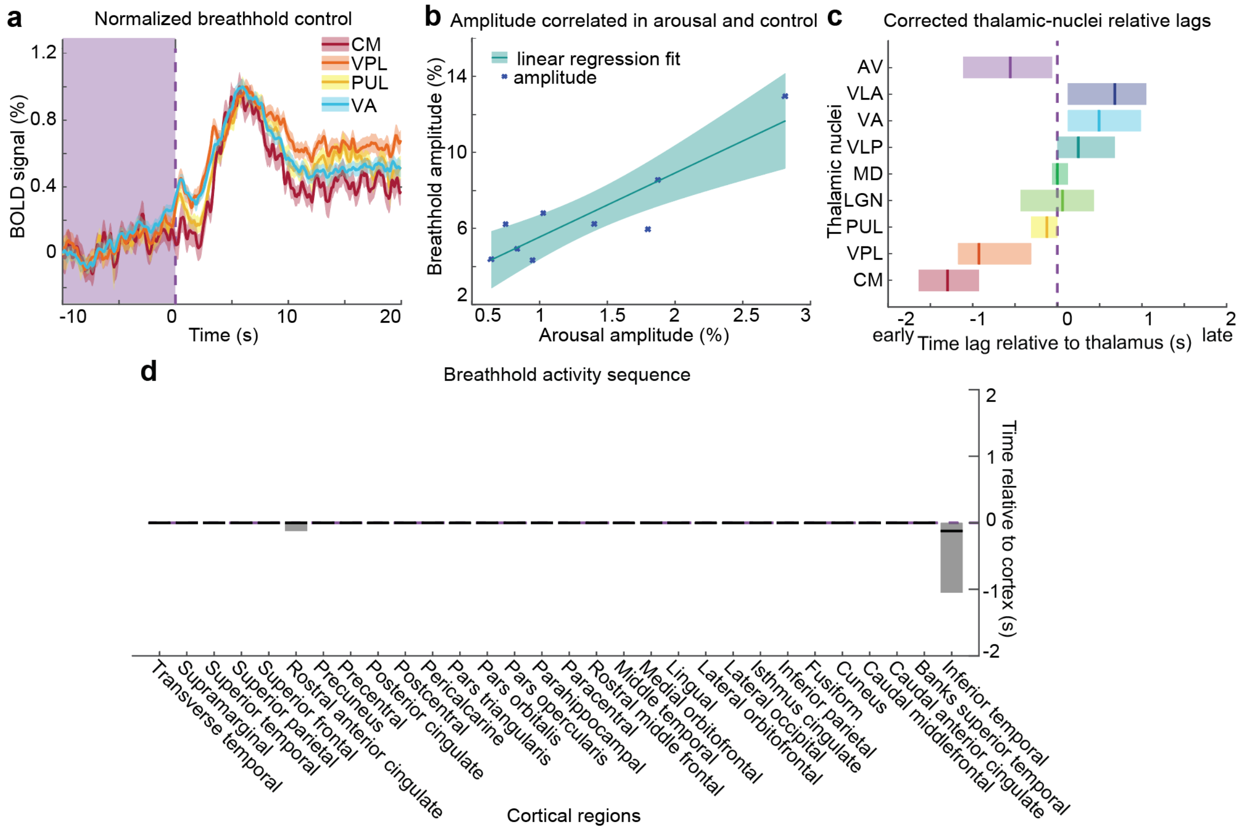

Supplementary Figure 11. Endogenous hemodynamics could not explain the observed thalamic nuclei sequence during behavioral arousal. a) Normalized signals from a subset of thalamic nuclei at breathhold release (purple dashed line). Data are presented as mean values and shading represents standard error. b) The amplitude of the breathhold response across thalamic nuclei was highly correlated with their amplitudes during behavioral arousal, suggesting successful recapitulation of local SNR and hemodynamic properties. c) Correcting the thalamic sequence by subtracting the average lag during breathhold release does not greatly alter the activation sequence, with VPL and CM still showing earlier activity. Color of each nucleus' lag represents order in original arousal sequence. The solid bar represents lag time. Shading represents the 95% confidence interval. d) No analogous activity sequence occurs across the cortex during the breathhold control that could explain the arousal-locked pattern. Source data are provided in "Fig 5 Source Data" file.

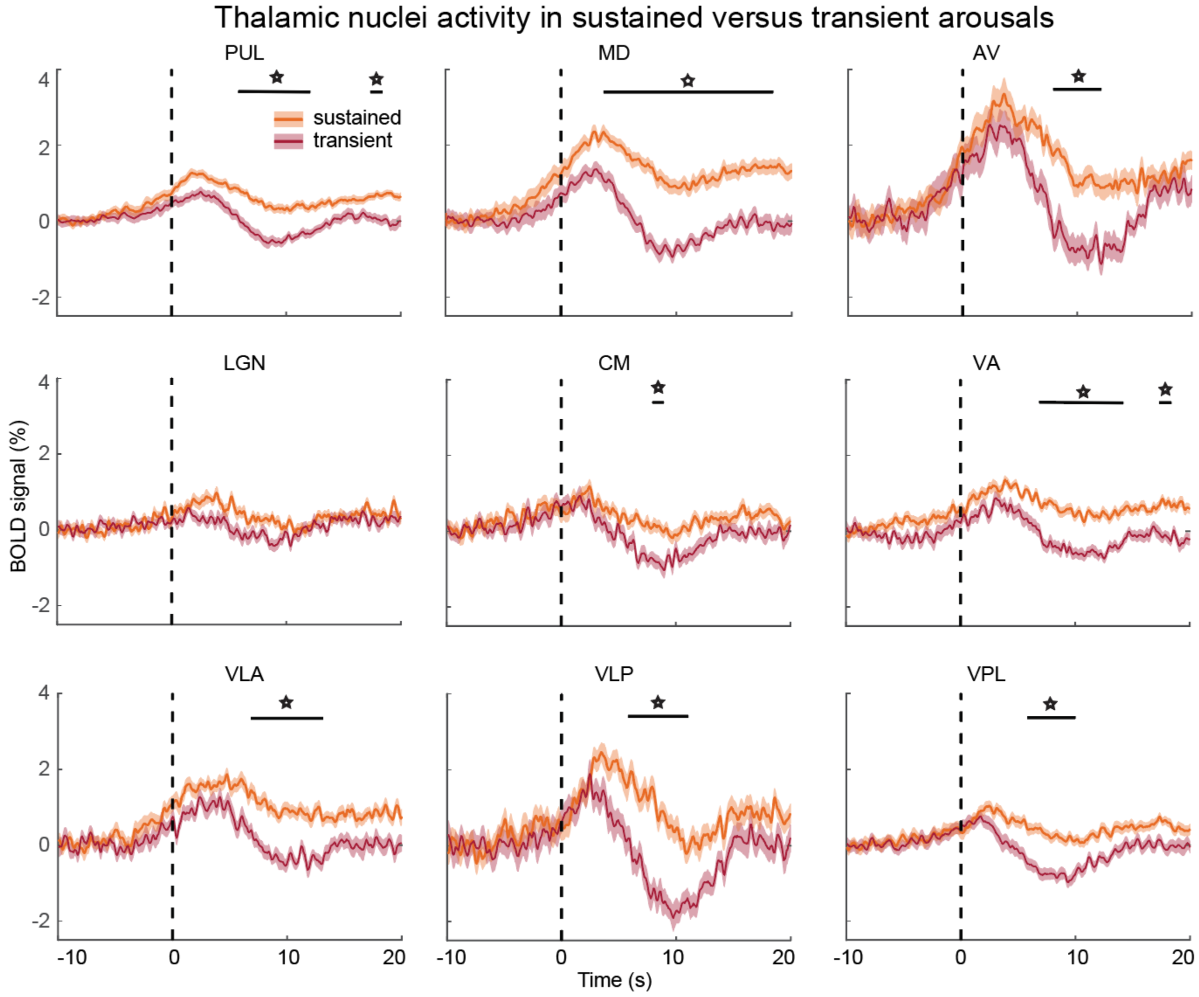

Supplementary figure 12: Thalamic activity at sustained vs transient arousals. Behavioral arousal is represented as the dashed vertical line. The response at sustained arousals is in orange, and at transient arousals is in red. Data are presented as mean values, and shading represents standard error. Most thalamic nuclei had significantly different post-arousal signals (starred horizontal line, $p<0.05$, Bonferroni corrected). Source data are provided in "Fig S12 Source Data" file.

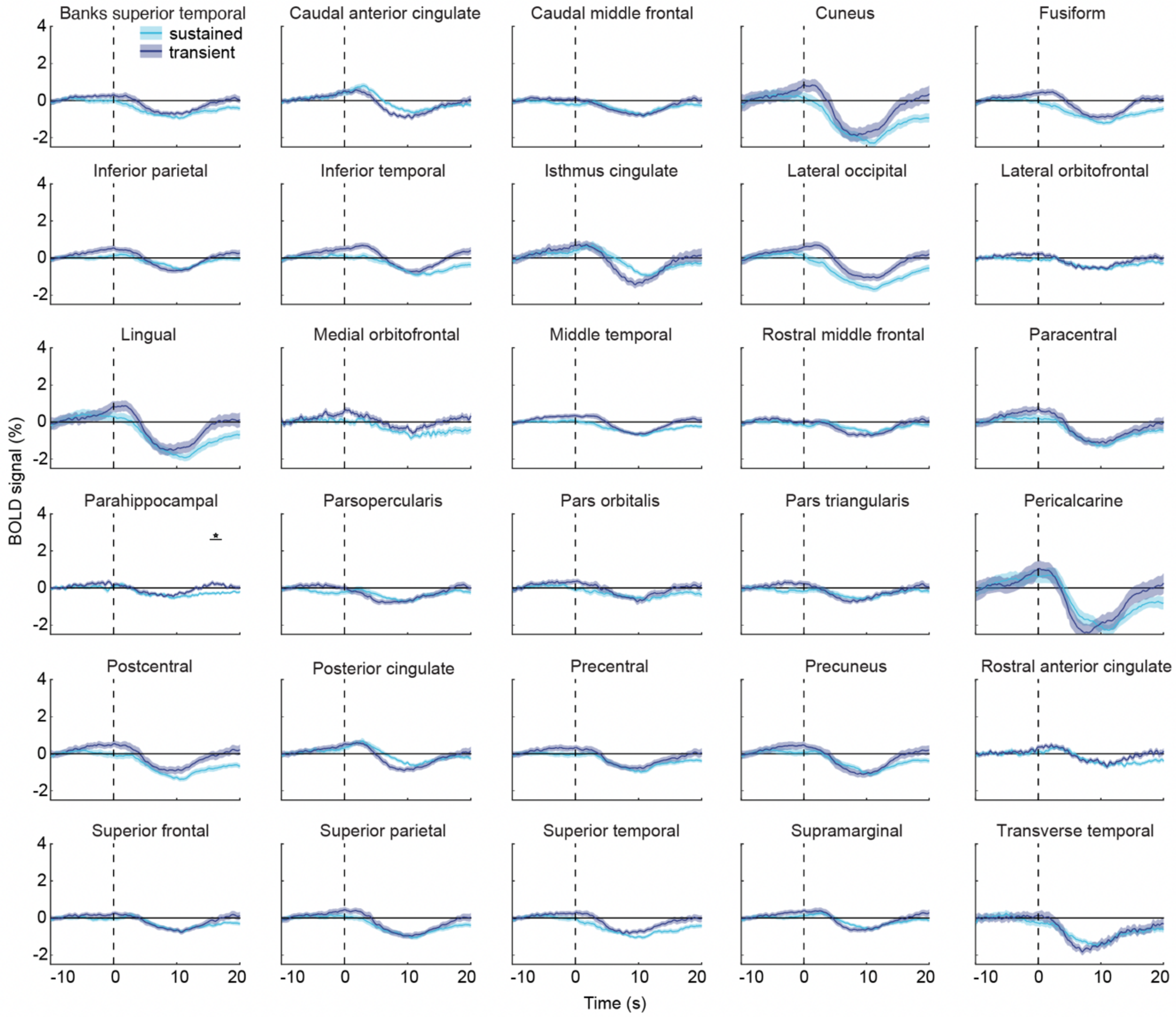

Supplementary figure 13: Cortical activity at sustained vs transient arousals. Behavioral arousal is represented as the dashed vertical line. The response at sustained arousals is in light blue, and at transient arousals is in dark blue. Data are presented as mean values, and shading represents standard error. Most cortical regions do not have significantly different BOLD signal in sustained and transient arousals (starred horizontal line, $p<0.05$, Bonferroni corrected). Source data are provided in "Fig 6 Source Data file".

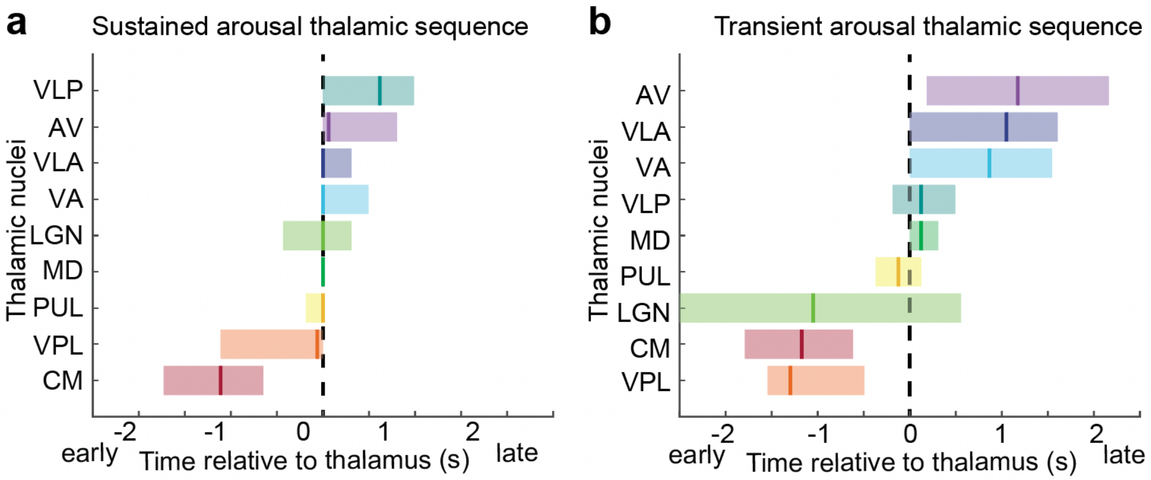

Supplementary figure 14: The activity sequence across thalamic nuclei differed in sustained (a) vs transient (b) arousals using a hierarchical bootstrap. Vertical line is the lag. Shaded rectangles represent the 95% confidence interval. Dashed black line represents the zero lag with the thalamus as a whole. Source data are provided in “Fig S14 Source Data file”.
