# Supplementary material for: A temporal sequence of thalamic activity unfolds at transitions in behavioral arousal state
Source: Nat Commun. 2022 Sep 16;13:5442. doi: 10.1038/s41467-022-33010-8 (PMC9481532; doi:10.1038/s41467-022-33010-8)
Supplement: Supplementary file 3 — Reporting Summary [file 41467_2022_33010_MOESM3_ESM.pdf]

nature portfolio

Corresponding author(s): Laura Lewis

Last updated by author(s): 10/2/2022

Reporting Summary

Nature Portfolio wishes to improve the reproducibility of the work that we publish. This form provides structure for consistency and transparency in reporting. For further information on Nature Portfolio policies, see our Editorial Policies and the Editorial Policy Checklist.

Statistics

For all statistical analyses, confirm that the following items are present in the figure legend, table legend, main text, or Methods section.

n/a	Confirmed	
☐	☒	The exact sample size (n) for each experimental group/condition, given as a discrete number and unit of measurement
☐	☒	A statement on whether measurements were taken from distinct samples or whether the same sample was measured repeatedly
☐	☒	The statistical test(s) used AND whether they are one- or two-sided<br>*Only common tests should be described solely by name; describe more complex techniques in the Methods section.*
☒	☐	A description of all covariates tested
☐	☒	A description of any assumptions or corrections, such as tests of normality and adjustment for multiple comparisons
☐	☒	A full description of the statistical parameters including central tendency (e.g. means) or other basic estimates (e.g. regression coefficient) AND variation (e.g. standard deviation) or associated estimates of uncertainty (e.g. confidence intervals)
☐	☒	For null hypothesis testing, the test statistic (e.g. F, t, r) with confidence intervals, effect sizes, degrees of freedom and P value noted<br>*Give P values as exact values whenever suitable.*
☒	☐	For Bayesian analysis, information on the choice of priors and Markov chain Monte Carlo settings
☒	☐	For hierarchical and complex designs, identification of the appropriate level for tests and full reporting of outcomes
☒	☐	Estimates of effect sizes (e.g. Cohen's d, Pearson's r), indicating how they were calculated

Our web collection on statistics for biologists contains articles on many of the points above.

Software and code

Policy information about availability of computer code

Data collection	We used EGI acquisition software version 5.4 to collect EEG data and LabChart version 7 to collect physiological data.
Data analysis	We used several free open source programs: AFNI version 19.1, FSL version 5, SPM version 12 in MATLAB 2020a,, Freesurfer developmental version from 8-12-2019, Fieldtrip version 20191025, and Chronux version 2.12. We used MATLAB built-in functions for data analysis.

For manuscripts utilizing custom algorithms or software that are central to the research but not yet described in published literature, software must be made available to editors and reviewers. We strongly encourage code deposition in a community repository (e.g. GitHub). See the Nature Portfolio guidelines for submitting code & software for further information.

Data

Policy information about availability of data

All manuscripts must include a data availability statement. This statement should provide the following information, where applicable:

- Accession codes, unique identifiers, or web links for publicly available datasets
- A description of any restrictions on data availability
- For clinical datasets or third party data, please ensure that the statement adheres to our policy

The mean region of interest time-series data locked to behavioral arousal are provided in the Supplementary Information/Source Data file. The raw behavioral arousal time-series across regions of interest are available in a public repository.

Field-specific reporting

Please select the one below that is the best fit for your research. If you are not sure, read the appropriate sections before making your selection.

☒ Life sciences ☐ Behavioural & social sciences ☐ Ecological, evolutionary & environmental sciences

For a reference copy of the document with all sections, see nature.com/documents/nr-reporting-summary-flat.pdf

Life sciences study design

All studies must disclose on these points even when the disclosure is negative.

Sample size	Since the effect size for this basic science study was not known in advance, we chose the number of subjects based on previous imaging studies of sleep. We had found that this number of subjects elicited robust neural effects during sleep in prior studies (Fultz 2019).
Data exclusions	EEG data was excluded if the subject did not present the occipital alpha rhythm during eyes closed wakefulness because this was the marker we used for wakefulness in the EEG. Individual behavioral arousals were excluded from analysis if there was more than 0.3 mm of motion in the 20 seconds before and after behavioral arousal. Subjects were excluded from analysis if they did not have any behavioral arousals.
Replication	We replicated our main finding of thalamocortical divergence at arousals in a second independent 3T dataset that is included in the manuscript.
Randomization	This doesn't apply to our study because we are not testing between groups.
Blinding	This doesn't apply to our study because we are not testing between groups.

Reporting for specific materials, systems and methods

We require information from authors about some types of materials, experimental systems and methods used in many studies. Here, indicate whether each material, system or method listed is relevant to your study. If you are not sure if a list item applies to your research, read the appropriate section before selecting a response.

Materials & experimental systems

n/a	Involved in the study	
☒	☐	Antibodies
☒	☐	Eukaryotic cell lines
☒	☐	Palaeontology and archaeology
☒	☐	Animals and other organisms
☐	☒	Human research participants
☒	☐	Clinical data
☒	☐	Dual use research of concern

Methods

n/a	Involved in the study	
☒	☐	ChIP-seq
☒	☐	Flow cytometry
☐	☒	MRI-based neuroimaging

Human research participants

Policy information about studies involving human research participants

Population characteristics	Participants in all datasets were screened not to have any neurological, psychiatric, or sleep disorders and not currently be taking psychiatric or sleep medications. Our analysis of Experiment 1 data used previously published data (Fultz et al., 2019) and included the subset of subjects who were instructed to perform a behavioral task during the sleep imaging (n=6; one male and five female), with a mean age of 24.6 years (range: 23–26).<br>The second dataset (Experiment 2) was a newly acquired imaging experiment using the same behavioral task, performed with fast fMRI at 7T. Written informed consent was obtained from 20 healthy adults (14 female and six male; mean age: 24.9, age range: 22–33).<br>For the third dataset (Experiment 3), eight subjects participated in the 7T fMRI breathhold control experiment (three females and five males, mean age=25.5, range: 20–34), with the same exclusion criteria as Experiment 2. Three of these individuals were also participants in Experiment 2. No participants were excluded.
Recruitment	Subjects were recruited through advertisements on the MGH website for open studies. Self-selection bias is that subjects are interested in participating in MRI studies. This is not expected to impact the results of the healthy young adult brain. No further replications were performed.
Ethics oversight	The Massachusetts General Hospital Institutional Review Board oversaw the ethics of this study.

Note that full information on the approval of the study protocol must also be provided in the manuscript.

Magnetic resonance imaging

Experimental design

Design type	event-related
Design specifications	We aimed to collect three 30-minute runs and did so if the subject remained comfortably sleeping in the scanner. The specific number of runs varied depending on how long the subject remained comfortably sleeping and if they pressed the button indicating a need to speak with experimenters.
Behavioral performance measures	This is fully reported in the Methods section. We asked our subjects to press a button on every breath in and out. Behavioral arousals were defined as the first button press after at least 20 seconds without activity.

Acquisition

Imaging type(s)	structural and functional
Field strength	7T
Sequence & imaging parameters	A T1-weighted anatomic multi-echo MPRAGE scan with 0.75 mm3 isotropic voxels was performed to provide an anatomical reference. Participants then underwent one to three EPI scans for fMRI data acquisition, containing up to 8000 volumes and lasting up to 33 minutes. 40 oblique slices were acquired with a voxel size of 2.5 mm3 isotropic, TE=24 ms, TR=247 ms, MultiBand factor=8, shift factor=4 matrix=84×84, flip angle=30°, echo spacing=0.53 ms, and no in-plane acceleration.
Area of acquisition	The area of acquisition covered most of the brain, but was positioned so that the thalamus and as much cortex as possible was captured without including the eyes since that can increase the noise level.

Diffusion MRI ☐ Used ☒ Not used

Preprocessing

Preprocessing software	Preprocessing of MRI data was performed using Freesurfer developmental version from 08-12-2019 (https://surfer.nmr.mgh.harvard.edu/fswiki) and FSL version 6 (https://fsl.fmrib.ox.ac.uk/fsl/fslwiki). Anatomical images were bias-corrected using SPM and then automatically segmented using Freesurfer. Functional images were realigned to correct motion artifacts using AFNI (https://afni.nimh.nih.gov/), and slice-time corrected using FSL.
Normalization	To preserve anatomical accuracy no cross subject image registration was used.
Normalization template	No normalization was used.
Noise and artifact removal	We used dynamic regression based on the concept of RETROICOR to remove signals driven by the heartbeat and respiration from the data while allowing the peak frequency of these physiological rhythms to vary over time. The cardiac signal was bandpass filtered between 0.2 and 10 Hz. Peaks of the cardiac signal were identified using the automated peak detection technique in the Chronux toolbox (http://chronux.org/), and the interpeak intervals were transformed into phases. The respiratory signal was bandpass filtered between 0.16–0.4 Hz using a finite impulse response filter, and the instantaneous phase was computed as the angle of the Hilbert transform. This phase information was transformed into sine functions, and beta values were estimated in a window of 1000 s sliding every 400 s voxel-wise using a general linear model. These values were then interpolated across each time-point and used to remove each voxel's first and second harmonic frequencies of the cardiac and respiratory signals.<br><br>We excluded behavioral arousals with more than 0.3 mm of motion as estimated during motion artifact correction by AFNI.
Volume censoring	No volume censoring was used.

Statistical modeling & inference

Model type and settings	A bootstrap analysis was used to resample with replacement from the arousals, generate a resampled mean timeseries, and calculate 95% confidence intervals of the lags between regions, resampling 1000 times. We also performed a heirarchical bootstrap analysis as a secondary analysis. First, we resampled the subjects with replacement and then resampled arousals with replacement from individual subjects. The mean fMRI signal during arousal for each ROI was computed, and the cross-correlation analysis was repeated. The upper and lower bounds of the 95% confidence interval of the lag between each nucleus and the whole thalamus were computed by calculating the 2.5 and 97.5 percentiles.<br><br>For onset time estimation, we fit a linear combination of two Gaussian curves to each ROI using a simple search method to find the parameters with the smallest root mean squared error. We defined onset time as the time that the model fit reached 10% of its maximum amplitude. In order to allow for temporal flexibility, we included the temporal derivative of each Gaussian in the model fit.
Effect(s) tested	We tested for significant arousal-locked activity change using two-sided t-tests, and we tested for significant differences in the timing of the activity change using a hierarchical bootstrap analysis.

Specify type of analysis: ☐ Whole brain ☒ ROI-based ☐ Both

Anatomical location(s)	Registration between the functional and anatomical images was completed using Freesurfer boundary-based registration. Cortical ROIs were extracted using the Desikan-Killiany atlas to identify functional voxels that were at least 70% filled by each region. The thalamic segmentation was done using the individual-level probabilistic atlas in the Freesurfer developmental version which provides voxel-wise segmentation probabilities in individual anatomical space, that have been previously validated against ex vivo histology. Nuclei were defined by selecting functional voxels that had at least a 90% chance of falling within a given thalamic nucleus to minimize partial-volume effects. All voxels that fell in any of the four pulvinar sub-regions were combined into a single pulvinar nucleus ROI, and similarly, and any voxels than fell in either of the two mediodorsal sub-regions were combined into the mediodorsal nucleus ROI. Cortical and thalamic regions which were not captured in every subject were excluded from the analysis.
Statistic type for inference (See Eklund et al. 2016)	No whole brain voxel-wise analyses were performed.
Correction	We corrected for multiple comparisons using the Bonferroni correction method in which we multiplied the p-value by the number of total tests run (number of ROIs and number of time points tested) before testing if the p-value was below 0.05.

Models & analysis

n/a	Involved in the study
☒	☐ Functional and/or effective connectivity
☒	☐ Graph analysis
☒	☐ Multivariate modeling or predictive analysis
